# Salt-Free Dyeing of Modified Cotton through Graft Polymerization with Highly Enhanced Dye Fixation and Good Strength Properties

**DOI:** 10.3390/polym12020462

**Published:** 2020-02-17

**Authors:** Wei Ma, Sen Du, Shumin Yan, Xiaolin Yu, Zhongjian Zhang, Shufen Zhang

**Affiliations:** State Key Laboratory of Fine Chemicals, Dalian University of Technology, No.2 Linggong Road, Gaoxinyuan District, Dalian 116023, Chinasara1995@mail.dlut.edu.cn (X.Y.); zhangshf@dlut.edu.cn (S.Z.)

**Keywords:** cotton fabric, modification, reactive dyes, graft copolymerization, salt-free dyeing

## Abstract

Modification of cotton fabric with 2-methacryloyloxyethyltrimethyl ammonium chloride (DMC) was achieved through free-radical initiated graft polymerization with K_2_S_2_O_8_/NaHSO_3_ as the initiator. Grafting of DMC was confirmed by ATR-IR of the modified cotton. The optimal grafting reaction conditions, including DMC dosage, mole ratio of initiator to DMC, temperature, and time, were determined by cation content and dye fixation results of the modified cotton. The modified fibers were characterized by X-ray diffraction (XRD), scanning electron microscope (SEM), and whiteness measurement. Salt-free dyeing of the modified cotton with commonly used C. I. Reactive Blue 19, C. I. Reactive Yellow 145, and C. I. Reactive Red 195 presented high fixation of 96.8%, 98.7%, and 97.3%, respectively. These results indicated that the modification is effective for changing the surface charge of the fiber and increasing the dye-fiber reactivity. The color fastness and strength property were still very satisfactory. With excellent properties, this dyeing method shows promise in real application for eliminating the usage of salt and reducing environmental pollution.

## 1. Introduction

Cotton fiber is one of the most popular natural fiber because of its softness, hygroscopicity, air permeability, and abrasion resistance performance [1,2,3]. For dyeing cotton fibers, reactive dyes are commonly used in virtue of their outstanding properties including brilliant color, excellent color fastness, wide range of hue, and convenient application [4,5]. Despite a short application history, the significant advantages make it possible that reactive dyes are the best choice for cotton dyeing to date.

However, the reactive dyeing industry now faces the unprecedented pressure of wastewater treatment for environmental protection. Utilization of reactive dyes in dyeing is only 50–80%, large amounts of hydrolyzed dyes are released and cannot be reused, which is a great waste; moreover, 30–100 g/L sodium sulphate or sodium chloride needs to be added in dye-bath to promote dye adsorption on cotton, the salt is not consumed during dyeing and discharged as waste after dyeing [1]. Plenty of rinsing water is used to wash off the hydrolyzed dyes and salt staining on fibers, which causes a big waste of water resource [6,7]. Therefore, in reactive dyeing, a large amount of colored wastewater containing salt is produced and is very difficult to deal with [8]. The above problems are all very pressing environmental issues to be solved.

Many attempts have been made to solve these problems, for example, manufacture of reactive dyes with high reactivity, production of cationic reactive dyes, modification of cotton, application of auxiliaries in dyebath, etc. [9,10,11,12]. Among them, modification of cotton is very important, it has received more and more attention in dyeing industry recently. Researchers found that cotton modification can not only change the surface charge of the fibers from negative to positive to promote dye adsorption, it also increases the reactivity of the fibers to achieve high dye fixation [13]. Chemical modification agents, such as glycidyltrimethylammonium chloride (GTA), 3-chloro-2-hydroxypropyltrimethylammonium chloride (CHPTAC), polyepichlorohydrin-dimethylamine (PECH-amine), and betaine, have been investigated to modify cotton [8,14,15,16]. These kinds of agents form stable chemical bonds with the fibers through etherification or esterification of the hydroxyl groups of the cellulose.

In recent years, graft polymerization has been widely studied for its ease of operation and good applicability [17,18,19,20]. Graft polymerization is also an effective way to chemically modify cotton [21,22,23]. Compared with other modification reactions, graft polymerization shows an advantage in that one grafting site can introduce more cationic groups, which is beneficial for achieving high cationic degree and promoting dye adsorption effectively. This method also shows the merit of not making the inherent advantages of cellulose being destroyed [24]. Textile grafting with different monomers can impart the fabric various properties and the market prospect is broad [25,26]; however, its application in salt-free dyeing of reactive dyes is seldom reported. In our previous study, we have investigated the ceric (IV) ion-initiated graft polymerization of cationic monomer onto cotton fabrics, and achieved high dye fixation without usage of inorganic salt. However, it was found that the fabrics turned a little yellow after graft polymerization mainly due to the orange color of the initiator—ammonium cerium (IV) nitrate. In addition, the tensile strength was decreased mainly attributed to glucose ring open during Ce (IV) initiated-graft polymerization in nitric acid as the cellulose backbone rather than the hydroxyl groups of the fibers is generally accepted as the major oxidation site [27]. 

**Figure 1:**
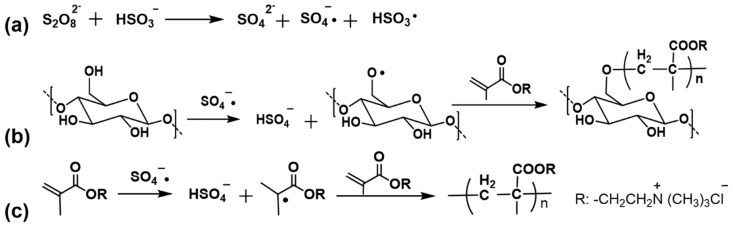
(**a**) Formation of free radicals; (**b**) reaction mechanism of grafting DMC onto cotton fiber in the presence of K_2_S_2_O_8_/NaHSO_3_; (**c**) homopolymerization of DMC.

In this paper, in order to utilize the advantages of graft polymerization and avoid the adverse effects of initiator Ce (IV), we designed to use redox initiator- K_2_S_2_O_4_/NaHSO_3_ to initiate graft polymerization of DMC onto cotton. The reaction mechanism and side reaction are shown in Figure 1. This redox initiator is water-soluble, and can initiate the graft polymerization under relatively low temperature in a short time [28,29]. As the initiator is colorless and the reaction conditions are mild, it will not cause color change of the fibers; moreover, as the radicals with this initiating system are generated on hydroxyl groups of the cellulose [30,31,32], graft polymerization shows much less influence on the strength properties of the fibers. As electro-withdrawing carbonyl groups are introduced to cellulose structure, the affinity between cotton and dyes may be improved to increase dye fixation. In this study, to achieve good dye fixation, the optimal graft polymerization conditions were investigated. Three commercial dyes C. I. Reactive Blue 19, C. I. Reactive Red 195, and C. I. Reactive Yellow 145 were employed to compare the dyeing properties of the modified cotton and the control one. The modified fibers were characterized, and the strength of the dyed cotton was also investigated to evaluate the application properties of this method.

## 2. Experimental

### 2.1. Materials

An aqueous solution of DMC (75% m/m) was purchased from Aladdin Chemical Reagent Co., Ltd., Shanghai, China. Potassium persulfate (K_2_S_2_O_3_) (99.5%) was obtained from Tianjin Guangfu Technology Development Co., Ltd. and sodium hydrogen sulfite (NaHSO_3_) was analytical reagent and purchased from Tianjin Damao Chemical Reagent Co., Ltd. (Tianjin, China). They were used as received without further purification. Bleached, desized, and mercerized cotton fabrics (150 g/m^2^) were purchased from Testfabric, Inc., Shanghai. Sodium sulphate and sodium carbonate were obtained from Tianjin Bodi Chemical Co., Ltd., Tianjin, China. Three reactive dyes used in this study are C. I. Reactive Blue 19, C. I. Reactive Yellow 145 and C. I. Reactive Red 195, they were supplied by Zhejiang Shunlong Chemical Co., Ltd. (Shaoxing, China).

### 2.2. Graft Polymerization of DMC onto Cotton

2.0 g of cotton fabric was immersed in an aqueous solution containing certain amount of DMC and K_2_S_2_O_4_/NaHSO_3_ (mass ratio of 1:0.4) in an Erlenmeyer flask. The flask was sealed, filled with nitrogen atmosphere, and then put in a dyeing machine (Xinwang Dyeing & Finishing Machinery Factory, Jingjiang, China). Graft polymerization was carried out at the temperature of 30 ± 0.1 °C at a liquor ratio of 20:1 for 60 min and the reaction system kept shaking for uniform modification. After that, the cotton fabric was taken out and rinsed with tap water several times to remove the unreacted monomers, and then air dried. 

### 2.3. Dyeing of Modified and Unmodified Cotton Fabrics

Both DMC-modified and unmodified cotton were dyed at a liquor-to-goods ratio of 20:1 using a laboratory dyeing machine (Xinwang Dyeing & Finishing Machinery Factory, Jingjiang, China). All the samples were dyed at 1.0%, 2.0%, and 3.0% o.w.f for C. I. Reactive Yellow 145, C. I. Reactive Red 195 and C. I. Reactive Blue 19, respectively, according to the parameters in industrial production.

Dyeing of the modified cotton commenced at 30 °C, and kept at the temperature for 30 min. Then the temperature was raised to 60 °C at 2 °C/min and 10 g/L of sodium carbonate was added to promote dye fixation. The dyeing process continued at 60 °C for 40 min before the fabric was removed off from the solution. Dyeing of the unmodified cotton was almost the same with that of the modified one except that 60 g/L sodium sulphate was added to the dye-bath in two batches at 30 °C. 

The dyed cotton fabrics were rinsed thoroughly in cold and warm water and soaped in 2 g/L washing detergent at 90 °C for 15 min, then the fabrics were washed in cold, warm, and cold water, then air dried. 

### 2.4. Degree of Substitution (DS) Measurement

*DS* of the cationic groups onto the modified fibers is one of the key parameters indicating the degree of graft copolymerization and it is calculated according to the following Equation (1)

(1) $$\mathrm{DS}\left(\mathrm{mmol}\cdot g^{-1}\right)=\frac{N\%\times 1000}{14.01}\times 100\%$$

where *N*% is the nitrogen content of the modified fiber obtained by the Kjeldahl method (GB12091-89) and 14.01 (g/mol) is the atomic weight of nitrogen. 

### 2.5. Characterization

The Fourier transform infrared (FTIR) spectra of the modified and unmodified fibers were recorded on a NICOLET 6700 FTIR spectrometer with a universal ATR sampling accessory from Thermo Fisher Scientific Co., Waltham, MA, USA. All samples were prepared as potassium bromide tablets. The X-ray diffraction (XRD) patterns of the modified and unmodified fibers were performed via an X-ray diffractometer (Rigaku D/MAX2400, Rigakub Co., Tokyo, Japan) using Cu Ka1 radiation. The surface morphologies of the modified and unmodified fibers were observed directly with scanning electron microscopy (SEM; JSM-5600LV, JEOL Co., Tokyo, Japan).

### 2.6. Color Measurement

Dye exhaustion (E%), fixation (F%), and reactivity (R%) are the basic parameters measuring the effects of dyeing and they are calculated according to Equations (2)–(4)

*E%* = (1 − *A*_1_/*A*_0_) × 100 (2)

*F%* = (*E%* − *A*_2_/*A*_0_) × 100 (3)

*R%* = (*E%*/*F%*) × 100 (4)

where *A*_0_, *A*_1_, and *A*_2_ are the absorbance of the original, dyeing residual, and soaping solution, respectively. All of the samples were tested under the maximum absorption wavelength (λ_max_) of the dyes via the UV–Vis spectrophotometer (Agilent HP8453, HP Co., Palo Alto, CA, USA) and calculated on the basis of the Lambert–Beer’s Law.

Color strength (K/S) was instrumentally determined using the Electronic Colour Matching Instrument UltraScanXE from Hunter Co., Reston, VA, USA, and it is proportional to the dye concentration on cotton.

### 2.7. Color Fastness

Wash fastness was tested using a S-1002 two-bath dyeing and testing apparatus (Roaches International Ltd., UK). Rub fastness was obtained using a Y (B) 571-II crockmeter (Wenzhou Darong Textile Instrument Co., Wenzhou, China) and the light fastness was tested with the light fastness tester (type 150s, Heraeous Co., Hanau, Germany). The wash, rub, and light fastness was evaluated by the ISO 105-E01-1995, ISO 105-X12-1993, and ISO105-B06-1998 standard, respectively.

### 2.8. Strength Testing

Tensile and tearing strength properties were evaluated using a YG(B)026H-250 fabric strength tester according to ISO13934.1-1999 and a YG(B)033 fabric tearing tester according to ISO 13937-1-2000, respectively. Both testers were from Wenzhou Darong Textile Instrument Company, Wenzhou, China.

## 3. Results and Discussion

### 3.1. Optimization of Preparation Conditions of the Modified Cotton Fibers

For grafting DMC onto cotton fibers, the very important step is to produce free radicals on the fibers. K_2_S_2_O_8_/NaHSO_3_ initiator produces free radicals through redox reaction, then it can activate the hydroxyl groups of the cellulose and form oxygen radicals on them, further initiate graft polymerization of monomer onto cellulose [33]. As the initiator exists in the reaction solution, formation of free radicals on monomers cannot be totally avoided. Reaction conditions determine the formation of free radicals and grafting ratio of DMC, and further influence dye adsorption and fiber reactivity. Therefore, the optimization of preparation conditions is essential for achieving high grafting ratio and dye exhaustion on the modified fibers in the absence of salt. 

Preparation parameters, including mole ratio of the initiator to DMC, reaction temperature, reaction time and DMC concentration were all optimized based on results of DS of the cationic group and the dye exhaustion (E%). 

**Figure 2:**
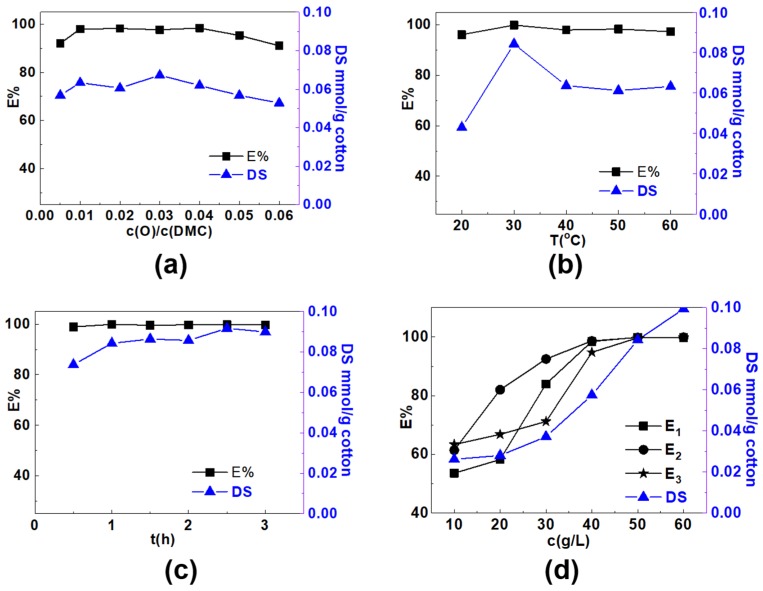
Influence of (**a**) mole ratio of K_2_S_2_O_8_ to DMC (concentration of DMC is 50 g/L, T = 40 °C and t = 3 h); (**b**) reaction temperature (concentration of DMC is 50 g/L, mole ratio of K_2_S_2_O_8_ to DMC is 0.01:1 and t = 3 h); and (**c**) reaction time (concentration of DMC is 50 g/L, mole ratio of K_2_S_2_O_8_ to DMC is 0.01:1 and T = 30 °C) on the DS of cotton and E% of C. I. Reactive Blue 19. (**d**) Influence of DMC concentration on DS and E% of C. I. Reactive Blue 19 (E_1_), C. I. Reactive Red 195 (E_2_) and C. I. Reactive Yellow 145 (E_3_) (mole ratio of K_2_S_2_O_8_ to DMC is 0.01:1, T = 30 °C and t = 1 h).

The influence of the mole ratio of K_2_S_2_O_8_ to DMC on DS and exhaustion of C. I. Reactive Blue 19 was investigated first (see Figure 2a). Concentration of DMC was kept at 50 g/L, and reaction temperature and time were set at 40 °C and 3 h, respectively. It showed with the increase of the mole ratio from 0.005 to 0.06, the DS first increased, and then decreased. When the ratio was 0.005, DS was 0.0568 mmol/g; when it increased to 0.03, DS reached the maximum value of 0.0673 mmol/g. Further increase in mole ratio resulted in DS decrease to some degree. It was found the change tendency of the dye exhaustion was almost the same with that of DS. At mole ratio ranging from 0.01 to 0.04, dye exhaustion was all high, 98% or higher; further increase of the mole ratio to 0.06, dye exhaustion decreased to 91.0% on the contrary. 

The above results demonstrate lower mole ratio is beneficial for graft polymerization. Initially, when the dosage of initiator is low, the active sites are far from enough for graft polymerization. With the increase of the concentration of free radicals, grafting ratio of DMC increased significantly. While at higher free radical concentration, homopolymerization among monomers in the solution increased [34,35,36]. Moreover, much initiator accelerated coupling of the free radicals, namely chain termination, which did not benefit increase of the degree of the graft polymerization [37]. As a consequence, at a higher mole ratio of initiator to DMC, both DS and dye exhaustion decreased.

The reaction temperature was examined as presented in Figure 2b and the other reaction conditions are: 50 g/L of DMC, 0.01:1 of c(O)/c(DMC) and 3 h of reaction time. The results showed that temperature had great effect on graft polymerization. When the temperature increased from 20 °C to 30 °C, DS increased from 0.0429 to 0.0843 mmol/g; when the temperature further increased, DS decreased on the contrary. The highest dye exhaustion of near 100% also appeared when the reaction temperature was 30 °C. It is reported higher temperature is not only good for chain initiation and transfer, it also benefits chain termination of graft polymerization; moreover, temperature increase also accelerates homopolymerization. Therefore, the graft ratio of DMC on cotton will be adversely affected at higher temperature. With the further increase of temperature, the pyrolysis of potassium persulfate at high temperature produced more free radicals, which alleviated the downward trend [38,39]. 

In addition, effect of reaction time on DS of cationic groups was also investigated at 30 °C with DMC of 50 g/L and c(O)/c(DMC) of 0.01:1. It was found from Figure 2c that when the reaction time increased from 0.5 to 1 h, the DS increased from 0.0737 to 0.0843 mmol/g, and dye exhaustion reached nearly 100% at 60 min. With further increase of the reaction time to 90, 120, 150 and 180 min, DS increased gradually, while dye exhaustion almost kept at 100%, indicating the cationic groups are enough for dye adsorption at reaction time of 60 min.

Besides the above conditions, influence of DMC concentration was also explored. It showed in Figure 2d, with the increase of the concentration of DMC from 10 to 60 g/L, DS of the cationic group almost showed linear increase and it increased from 0.0262 gradually to 0.0994 mmol/g. The increase in monomer concentration could increase the driving force of concentration gradient and accelerate the formation of graft copolymer. It presents in Figure 2d, with the increase of DMC dosage from 10 to 40 g/L, exhaustion (E_1_) of C. I. Reactive Blue 19 increased very quickly from 53.6% to 98.5%; when DMC further increased to 60 g/L, growth of E_1_ slowed down, and finally reached 99.8%. For C. I. Reactive Red 195 (E_2_) and C. I. Reactive Yellow 145 (E_3_), quick dye exhaustion increase was also observed when DMC increased from 10 to 40 g/L, further increase to 50 g/L, dye exhaustion could reach near 100% for both dyes, indicating sufficient cationic sites for adsorption of the anionic dyes.

The above results demonstrated introduction of cationic groups onto cotton fabrics promoted dye adsorption effectively, and dye exhaustion could reach an extremely high value, nearly 100%, which is far higher than that obtained in the conventional dyeing with 60 g/L of salt addition, revealing effectiveness of this modification method in achieving high dye exhaustion. 

Based on the study of parameters for graft copolymerization, the optimized preparation conditions were concluded. With DMC concentration of 50 g/L, mole ratio of K_2_S_2_O_8_ to DMC of 0.01:1, reaction temperature of 30 °C, and time of 60 min, all three dyes could reach near 100% dye exhaustion.

### 3.2. Characterization of Cationic Fibers

#### 3.2.1. FTIR Analysis of the Modified Cotton

**Figure 3:**
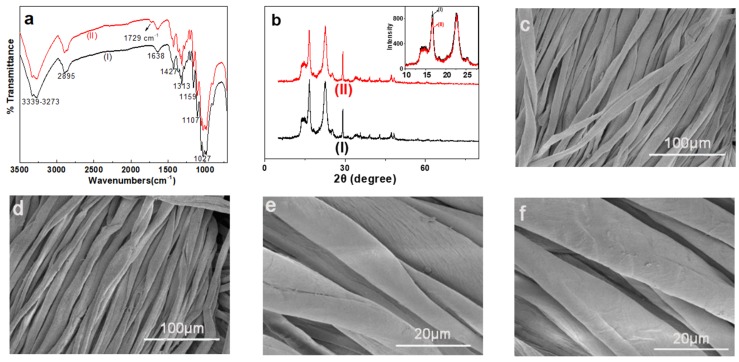
(**a**) ATR-IR spectra of the original (I) and the modified cotton fibers (II); (**b**) X-ray diffraction patterns of the original (I) and the modified cotton fibers (II); (**c**,**e**) SEM images of the original cotton fibers and (**d**,**f**) SEM images of the modified cotton fibers ((**c**,**d**) ×1000, (**e**,**f**) ×5000).

Modification of the cotton fibers through graft polymerization was confirmed by ATR-IR spectrum, and the results were shown in Figure 3a. Before IR measurement, the modified cotton was washed thoroughly to remove the ungrafted water-soluble monomer and homopolymer. In the spectrum of the original cotton fibers, it appeared stretching vibration peaks of O-H and methylene C-H at 3273 cm^−1^ and 2895 cm^−1^, respectively. Moreover, bending vibration peak of O-H appeared at 1638 cm^−1^, and deformation vibration peaks of methylene C-H and methine C-H at 1427 cm^−1^ and 1313 cm^−1^, respectively. In addition, the peak at 1027 cm^−1^ was assigned to stretching vibration of C-O-C. All these are characteristic bands of cotton fibers. Compared with the IR spectrum of the original cotton fibers, IR spectrum of DMC-modified one exhibited a distinct additional absorption peak at 1729 cm^−1^, which is attributed to the stretching vibration of ester carbonyl group of DMC. Therefore, IR spectrum demonstrated that DMC has been grafted onto cotton through graft polymerization [40].

#### 3.2.2. XRD Analysis

Figure 3b shows the XRD of the original and modified fibers for investigation of cotton crystallinity. It can be seen from the figure that the crystalline form of both original and modified fibers is cellulose I, whose XRD pattern mainly exhibited three peaks at 2*θ* = 14.8°, 16.6°, and 22.6°. Therefore, under the graft polymerization conditions, the crystalline form of the cellulose did not change, indicating most grafting occurred on cotton surface and in the amorphous region. In the inserted enlarged figures of Figure 3b, it can be clearly observed that the peak height is almost the same after modification, indicating almost no influence of the grafting on the crystallinity of the modified fibers.

#### 3.2.3. SEM Analysis

SEM was used to visually observe the change in surface morphology of the cotton fibers before and after grafting polymerization, and the results were displayed in Figure 3c–f.

Figure 3c,e are the SEM images of the original cotton fibers magnified 1000 and 5000 times, respectively; Figure 3d,f are the ones of the corresponding modified ones. Comparison of Figure 3c with Figure 3d, and Figure 3e with Figure 3f was made and almost no obvious difference could be observed; only slight rougher surface of the fibers was found after graft polymerization. Based on the above results, it can be concluded that the surface morphology before and after modification was basically the same.

#### 3.2.4. Colorimetric Properties

The color, whiteness and yellowness of the original and modified cotton were tested and compared. The results were shown in Table 1.

The results showed that the whiteness of the original and modified cotton are 81.0 and 81.5, respectively, indicating that the modified one was even whiter; yellowness of the modified cotton was measured to be 1.1, while that of the original one was 1.4, which also demonstrated the DMC-treated fibers was less yellow. Much whiter modified cotton is mainly due to the removal of the impurity from the cotton surface. Therefore, the graft polymerization with K_2_S_2_O_8_/NaHSO_3_ as initiator did not show any adverse influence on the color of the cotton, it could even make the cotton whiter.

#### 3.2.5. Application Performance

**Figure 4:**
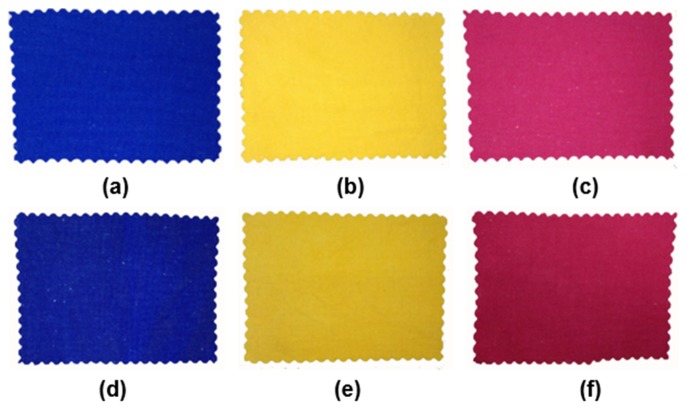
Dyeing samples of C. I. Reactive Blue 19, C. I. Reactive Yellow 145 and C. I. Reactive Red 195 on original and modified cotton fabrics: (**a**–**c**) on original cotton with the conventional dyeing method, (**d**–**f**) on modified cotton with the salt-free dyeing method.

The dyeing properties of C. I. Reactive Blue 19, C. I. Reactive Yellow 145 and C. I. Reactive Red 195 were studied and the results were shown in Figure 4 and Table 2. It can be seen from Figure 4 that the color depth of the modified cotton in salt-free dyeing process is significantly increased.

It exhibited in Table 2 that E%, F%, R%, and K/S values of the three dyes on the modified cotton were all improved significantly compared with that on the unmodified one in the presence of 60 g/L Na_2_SO_4_.

In Table 2, all dye exhaustion was higher than 99.0% and the reactivity was higher than 96.0%. The dye fixation of C. I. Reactive Blue 19, C. I. Reactive Yellow 145 and C. I. Reactive Red 195 reached 96.8%, 98.7%, and 97.3%, respectively, which was 29.6%, 22.9%, and 18.1% higher than those of the conventional dyeings, respectively. Due to introduction of cationic groups to cotton, the anionic dyes could effectively adsorb on cotton to increase dye exhaustion, and the adsorbed dyes have more opportunity to interact and react with the hydroxyl groups to further increase the dye fixation. For the color fastness tests, it showed the results on the DMC-treated cotton were all comparable with that on the untreated one, indicating the dyes were covalently bonded to the fibers. The light fastness of the dyes was even higher on the modified cotton, which was probably due to higher color depth. With this method, utilization of the reactive dyes could be greatly enhanced and inorganic salt could be eliminated in dyeing. Therefore, the dyeing wastewater never contains large amounts of hydrolyzed dyes and salt as before, which definitely benefits the environment a great deal and saves costs for wastewater treatment.

#### 3.2.6. Strength Testing

Mechanical properties, including tensile and tear strength of the DMC-modified cationic cotton, were measured as shown in Table 3 and the results were compared with those of the untreated one. As exhibited in the table, the tensile strengths in warp and weft of the original fibers were 536.2 and 523.5 N, respectively. While those of the cationic fibers were 496.0 and 482.4 N, respectively. The decreases in warp and weft for the modified fibers were 2.4% and 2.7%, respectively. While the decreases in warp and weft when ceric (IV) ion was used as initiator were 4.5% and 4.2%, respectively [27], showing advantages of this modification method in maintaining the mechanical strength of the cotton fibers. Moreover, for tear strength in warp, only 1.8% decrease was obtained and that in weft was unchanged, indicating the influence of the graft-polymerization on tear strength was much less. The strength decrease was mainly due to chemical reactions between DMC and the hydroxyl groups of the cotton fibers, which weakened hydrogen bonding among the fibers, resulting in a slightly lower mechanical strength.

In addition, the strength properties of the dyed cotton fabrics were also investigated as displayed in Table 4. From the table, it showed dyeing with reactive dyes decreased both the tensile and tearing strengths of the cotton fibers as chemical reactions occurred during dyeing process. Graft polymerization of cotton showed certain adverse influence on tensile strength in warp for C. I. Reactive Blue 19 and C. I. Reactive Yellow 145, while those dyed with C. I. Reactive Red 195 did not show obvious difference. For the tensile strength in weft, the modified cotton even exhibited higher values, this is probably due to the dye filling effect resulted from higher dye fixation. Besides, it was found that the effect of cotton modification on tearing strength was relatively small for the dyed cotton. Therefore, from the data analysis, we can conclude that compared with the dyed unmodified one, the modified cotton showed a little decrease in warp of tensile strength, certain increase in weft, and also a little increase in both warp and weft of tearing strength. On the whole, this kind of cotton modification method is beneficial for maintaining the mechanical strength of the fabrics.

## 4. Conclusions

Through graft copolymerization with K_2_S_2_O_8_/NaHSO_3_ as initiator, cotton fabrics were effectively cationized by DMC. The ATR-IR spectrum of modified fibers showed successful grafting, and the cotton turned to be whiter in color after graft copolymerization. The optimal cation content of cotton was determined to be 0.0843 mmol/g at the condition of 50 g/L DMC, mole ratio of K_2_S_2_O_8_ to DMC of 0.01:1, and 30 °C for 60 min. Under the conditions, the three commercial dyes C. I. Reactive Blue 19, C. I. Reactive Yellow 145, and C. I. Reactive Red 195 reached fixation of 96.8%, 98.7%, and 97.3%, respectively, which increased by 29.6%, 22.9%, and 18.1%, respectively, compared with that obtained in conventional dyeing. All the experimental data indicated that the reactivity between cotton and dyes was improved with the addition of DMC. The color fastness of the dyes on the modified cotton was all satisfactory. Graft polymerization with K_2_S_2_O_8_/NaHSO_3_ as an initiator still showed a little adverse effect on strength properties of cotton. However, compared with ceric (IV) ion initiator, the influence was much lower. After dyeing, the change in tensile and tearing strength of the modified and the unmodified cotton was also compared, it showed this kind of cotton modification method is beneficial for maintaining the mechanical strength of the fibers. All these results showed a good application prospect of this dyeing method.

## Figures and Tables

**Table 1:** Colorimetric properties, whiteness, and yellowness of the original and modified cotton

Cotton	K/S	L*	a*	b*	Whiteness	Yellowness
Original	0.1	94.2	−0.8	0.8	81.0	1.4
Modified	0.1	94.1	−0.7	0.7	81.5	1.1

L*: brightness; a*: red–green value; b*: yellow–blue value.

**Table 2:** Dyeing properties of DMC-modified and original cotton

Dyes	No.	E%	R%	F%	K/S	Wash Fastness			Rub Fastness		Light Fastness
						Staining		Change	Dry	Wet	
						Cotton	Wool				
C. I. Reactive Blue 19	1	99.9	96.9	96.8	11.5	4–5	3	4	4–5	4	7
	2	81.1	82.8	67.2	9.7	4–5	3	4	4–5	4	6–7
C. I. Reactive Yellow 145	1	99.8	98.9	98.7	4.2	4–5	4–5	4–5	4–5	4	5–6
	2	96.7	78.4	75.8	3.7	4–5	4	4–5	4–5	4	4–5
C. I. Reactive Red 195	1	99.7	97.6	97.3	11.3	4–5	4	4–5	4	3	3–4
	2	91.0	87.0	79.2	8.5	4–5	4	4	4	3–4	2–3

1: DMC modified cotton, salt-free dyeing; 2: original cotton, conventional dyeing.

**Table 3:** Mechanical strength of the original and DMC-modified cotton

Cotton	Tensile Strength/N		Tearing Strength/N	
	Warp	Weft	Warp	Weft
Original (*N*_0_)	536.2	496.0	11.2	10.6
Modified (*N*_1_)	523.5	482.4	11.0	10.6
Dec%	−2.4	−2.7	−1.8	0

Dec% = (*N*_0_ − *N*_1_)/*N*_0_ × 100%.

**Table 4:** Mechanical strength of the original and modified cotton dyed with reactive dyes

Cotton		Tensile Strength /N		Tearing Strength /N	
		Warp	Weft	Warp	Weft
Undyed and original		536.2	496.0	11.2	10.6
Dyed with C. I. Reactive Blue 19	1	518.7	481.3	11.1	10.2
	2	528.7	472.2	10.6	10.1
Dyed with C. I. Reactive Yellow 145	1	513.4	489.9	10.9	10.9
	2	535.8	482.0	10.8	10.2
Dyed with C. I. Reactive Red 195	1	519.0	489.3	10.8	10.6
	2	514.9	473.2	10.7	10.2

1: Modified cotton with salt-free dyeing method; 2: original cotton with conventional dyeing method.

## References

[1] Wang Q.Q., Liu F., Chen X.S., Ma X.J., Zeng H.Q., Yang Z.M. (2010). Transcriptome profiling of early developing cotton fiber by deep-sequencing reveals significantly differential expression of genes in a fuzzless/lintless mutant. Genomics.

[2] Muresan E.I., Balan G., Popescu V. (2013). Durable hydrophobic treatment of cotton fabrics with glycidyl stearate. Ind. Eng. Chem. Res..

[3] Bhuiyan M.A., Hossain M.A., Zakaria M., Islam M.N., Uddin M.Z. (2016). Chitosan coated cotton fiber: Physical and antimicrobial properties for apparel use. J. Polym. Environ..

[4] Samanta A.K., Sharmistha C., Guha R.T. (2012). Dyeing of jute with reactive dyes: Optimisation of the process variables and assessment of colourfastness characteristics. J. Inst. Eng. (India) Ser. E.

[5] Rizk H.F., Ibrahim S.A. (2015). Synthesis, fastness properties, color assessment and antimicrobial activity of some azo reactive dyes having pyrazole moiety. Dyes Pigment.

[6] Adeyemo A.A., Adeoye I.O., Bello O.S. (2017). Adsorption of dyes using different types of clay: A review. Appl. Water Sci..

[7] Han G., Chung T.S., Weber M., Maletzko C. (2018). Low-pressure nanofiltration hollow fiber membranes for effective fractionation of dyes and inorganic salts in textile wastewater. Environ. Sci. Technol..

[8] Acharya S., Abidi N., Rajbhandari R., Meulewaeter F. (2014). Chemical cationization of cotton fabric for improved dye uptake. Cellulose.

[9] Hamaky Y.H., Tawfeek S., Ibrahim D.F., Maamoun D., Gaber S. (2010). Printing cotton fabrics with reactive dyes of high reactivity from an acidic printing paste. Color. Technol..

[10] Zhao T., Sun G., Song X.Y. (2010). An antimicrobial cationic reactive dye: Synthesis and applications on cellulosic fibers. J. Appl. Polym. Sci..

[11] Fu S., Hinks D., Hauser P., Ankeny M. (2013). High efficiency ultra-deep dyeing of cotton via mercerization and cationization. Cellulose.

[12] Wang G.W., Zhuang L.H., Sun J., Zheng C.L. (2014). Salt-free dyeing of ramie fabric with an amino-terminated hyperbranched polymer. Cellulose.

[13] Siddiqua U.H., Ali S., Iqbal M., Hussain T. (2017). Relationship between structures and dyeing properties of reactive dyes for cotton dyeing. J. Mol. Liq..

[14] Li R., Gu F. (2010). Dyeing properties of PECH-amine cationized cotton with acid dyes. J. Appl. Polym. Sci..

[15] Boonroeng S., Srikulkit K., Xin J.H., Liang H. (2015). Preparation of a novel cationic curcumin and its properties evaluation on cotton fabric. Fiber. Polym..

[16] Ma W., Meng M., Yan S., Zhang S. (2016). Salt-free reactive dyeing of betaine-modified cationic cotton fabrics with enhanced dye fixation. Chin. J. Chem. Eng..

[17] Garcia-Valdez O., Champagne P., Cunningham M.F. (2018). Graft modification of natural polysaccharides via reversible deactivation radical polymerization. Prog. Polym. Sci..

[18] Zhang X., Liu C., Zhang A., Sun R. (2017). Synergistic effects of graft polymerization and polymer blending on the flexibility of xylan-based films. Carbohydr. Polym..

[19] Olad A., Zebhi H., Salari D., Mirmohseni A., Tabar A.R. (2018). Slow-release npk fertilizer encapsulated by carboxymethyl cellulose-based nanocomposite with the function of water retention in soil. Mater. Sci. Eng. A.

[20] Zhang X., Wang H., Liu C., Zhang A., Ren J. (2017). Synthesis of thermoplastic xylan-lactide copolymer with amidine-mediated organocatalyst in ionic liquid. Sci. Rep..

[21] Sekine A., Seko N., Tamada M., Suzuki Y. (2010). Biodegradable metal adsorbent synthesized by graft polymerization onto nonwoven cotton fabric. Radiat. Phys. Chem..

[22] Ou K., Wu X., Wang B., Meng C., Dong X., He J. (2017). Controlled in situ graft polymerization of dmaema onto cotton surface via si-arget atrp for low-adherent wound dressings. Cellulose.

[23] Wang C.X., Ren Y., Lv J.C., Zhou Q.Q., Ma Z.P., Qi Z.M. (2017). In situ synthesis of silver nanoparticles on the cotton fabrics modified by plasma induced vapor phase graft polymerization of acrylic acid for durable multifunction. Appl. Surf. Sci..

[24] Xia D., Bao H., Ou K., Yao J., Wei Z., He J. (2015). Polymer-grafted modification of cotton fabrics by si-arget atrp. Fiber. Polym..

[25] Princia E., Pedemonte E., Gentile G., Cocca M., Martuscelli E. (2006). Synthesis and mechanical characterisation of cellulose based textiles grafted with acrylic monomers. Eur. Polym. J..

[26] Princi E., Vicini S., Pedemonte E., Arrighi V., Mcewen I.J. (2010). New polymeric materials for paper and textiles conservation. II. Grafting polymerization of ethyl acrylate/methyl methacrylate copolymers onto linen and cotton. J. Appl. Polym. Sci..

[27] Ma W., Wang T., Li H., Zhang S. (2015). Cotton fabric modification through ceric (IV) ion-initiated graft copolymerisation of 2-methacryloyloxyethyltrimethyl ammonium chloride to enhance the fixation of reactive dyes. Cellulose.

[28] Xue J.Q., Wang Y.J., Du Y.W., Xue Y.F., Wen D.D. (2011). Synthesis and characterization of modified chitosan by graft polymerization. Adv. Mater. Res..

[29] Jin E., Reddy N., Zhu Z., Yang Y. (2015). Graft polymerization of native chicken feathers for thermoplastic applications. J. Agric. Food Chem..

[30] Sabaa M.W., Mokhtar S.M. (2002). Chemically induced graft copolymerization of itaconic acid onto cellulose fibers. Polym. Test.

[31] Suo A., Qian J., Yu Y., Zhang W. (2010). Synthesis and properties of carboxymethyl cellulose-graft-poly(acrylic acid-co-acrylamide) as a novel cellulose-based superabsorbent. J. Appl. Polym. Sci..

[32] Xiu H.J., Han Q., Wang L.J., Zhang R. (2011). Effect of different initiator systems on graft copolymerization of cellulose fiber with caprolactam. Adv. Mater. Res..

[33] Storsberg J., Ritter H. (2015). Cyclodextrins in polymer synthesis: Free radical polymerization of cyclodextrin host-guest complexes of methyl methacrylate or styrene from homogenous aqueous solution. Macromol. Rapid Commun..

[34] Mostafa T.B., Naguib H.F., Sabaa M.W., Mokhtar S.M. (2010). Graft copolymerization of itaconic acid onto chitin and its properties. Polym. Int..

[35] Teli M.D., Sheikh J. (2012). Antibacterial and acid and cationic dyeable bamboo cellulose (rayon) fabric on grafting. Carbohydr. Polym..

[36] Teli M.D., Sheikh J. (2012). Graft copolymerization of acrylamide onto bamboo rayon and fiber dyeing with acid dyes. Iran. Polym. J..

[37] Sarbu T., Lin K.Y., Spanswick J. (2004). Synthesis of hydroxy-telechelic poly(methyl acrylate) and polystyrene by atom transfer radical coupling. Macromolecules.

[38] Sabaa M.W., Mohamed N.A., Mohamed R.R. (2011). Chemically induced graft copolymerization of 4-vinyl pyridine onto carboxymethyl chitosan. Polym. Bull..

[39] Singha A.S., Guleria A., Rana R.K. (2013). Ascorbic acid/H_2_O_2_-initiated graft copolymerization of methyl methacrylate onto abelmoschus esculentus fiber: A kinetic approach. Int. J. Polym. Anal. Charact..

[40] Sayyah S.M., Khaliel A.B., Mohamed E.H. (2013). Enhancing water resistance of paper by graft copolymerization reaction. J. Appl. Polym. Sci..

